# HEW-Score – ein Werkzeug zur Homogenisierung der Spendermeldungen an die DSO: Multizentrische retrospektive Analyse dreier Universitätsklinika

**DOI:** 10.1007/s00063-024-01237-6

**Published:** 2025-01-28

**Authors:** Felix Lehmann, Stefan F. Ehrentraut, Jan Görtzen-Patin, Martin Söhle, Juliane Langer, Mohammed Banat, Daniel Schrader, Holger Kraus, Johannes Weller

**Affiliations:** 1 Klinik für Anästhesiologie und Operative Intensivmedizin, Universitätsklinikum Bonn, Venusberg-Campus 1, 53127 Bonn, Deutschland; 2 Klinik für Innere Medizin I, Universitätsklinikum Bonn, Bonn, Deutschland; 3 Transplantationsbeauftragte, Stabsstelle Ärztliche Direktion, Universitätsklinikum Bonn, Bonn, Deutschland; 4 Klinik für Neurochirurgie, Universitätsklinikum Bonn, Bonn, Deutschland; 5 Stabsstelle Organspendekoordination, Universitätsklinik Düsseldorf, Düsseldorf, Deutschland; 6 Transplantationsbeauftragter, Universitätsmedizin Essen, Essen, Deutschland; 7 Klinik für vaskuläre Neurologie und Klinik für Neuroonkologie, Zentrum für Neurologie, Universitätsklinikum Bonn, Bonn, Deutschland

**Keywords:** Hirntod, Irreversibler Hirnfunktionsausfall, Neurointensivmedizin, Organspende, Spenderidentifikation, Brain death, Irreversible loss of brain function, Neurointensive care, Organdonation, Donor identification

## Abstract

**Hintergrund:**

Die anhaltend niedrige Anzahl postmortaler Organspenden in Deutschland führte wiederholt zu politischen Diskussionen und zuletzt zur Novellierung des Transplantationsgesetzes mit Stärkung der Rolle des Tansplantationsbeauftragten und Einführung eines Registers zur Dokumentation des Spendewillens. Hintergrund dieser Entscheidungen war die Annahme, dass ein relevanter Anteil potenzieller Organspender in den Krankenhäusern übersehen werden. Aufgrund fehlender Vorgaben, wann ein potenzieller Organspender an die DSO („Deutsche Stiftung Organspende“) gemeldet werden muss, ist die bestehende Datenlage jedoch nur von eingeschränkter Aussagekraft.

**Ziel der Arbeit:**

Die Transplantationsbeauftragten der Universitätsklinika in NRW verständigten sich daher auf den hier vorgestellten Hirnfunktionsausfall-Eignung-Wille(HEW)-Score als Standard für die Meldung potenzieller Organspender.

**Material und Methoden:**

Durch Zuordnung der Punktwerte von 1–3 zu jedem der 3 inkludierten Merkmale ergibt sich ein Score von 111–333 und ab Überschreitung des Schwellenwerts 213 wird eine Meldung an die DSO empfohlen. Zur Implementierung wurden retrospektiv die HEW-Scores der durch TransplantCheck ermittelten Fälle der Universitätsklinika Bonn, Essen und Düsseldorf aus 2022 erhoben und in dieser Arbeit dargestellt.

**Ergebnisse:**

Insgesamt lag die Anzahl der gemäß HEW-Score zu meldenden Fälle in allen 3 Standorten 13,5 % unter den tatsächlich gemeldeten Fällen (126 vs. 109). In allen 3 Standorten war die Ablehnungsquote mit 54,5–64,9 % hoch.

**Diskussion:**

Zusammenfassend stellt der HEW-Score ein Werkzeug zur detaillierten Erfassung und standardisierten Meldung potenzieller Organspender dar und kann ein homogenisiertes Meldeverhalten als Datengrundlage für zukünftige Verbesserungsansätze ermöglichen.

**Zusatzmaterial online:**

Zusätzliche Informationen sind in der Online-Version dieses Artikels (10.1007/s00063-024-01237-6) enthalten.

## Abstract (en)

**Background:**

The persistently low number of postmortem organ donations in Germany has repeatedly led to political discussions and most recently to the amendment of the Transplantation Act with the strengthening of the role of the transplantation officer and the introduction of a register to document the will to donate. The background to these decisions was the assumption that a relevant proportion of potential organ donors in hospitals were being overlooked and not reported. However, due to the lack of guidelines as to when a potential organ donor must be reported to the DSO (“Deutsche Stiftung Organspende”), the existing data is only of limited validity.

**Objectives:**

The transplantation officers of the university hospitals in North Rhine–Westphalia (NRW) therefore agreed on the HEW score (brain function loss–suitability–will) presented here as a shared standard for reporting potential organ donors.

**Materials and methods:**

Assigning the scores from 1 to 3 to each of the three included attributes results in a score of 111 to 333, and reporting to the DSO is recommended if the threshold value of 213 is exceeded. For implementation, the HEW scores of the cases determined by TransplantCheck from the university hospitals in Bonn, Essen and Düsseldorf from 2022 were collected retrospectively and presented in this paper.

**Results:**

Overall, the number of cases to be reported according to the HEW score was 13.5% below the number of cases actually reported at all three sites (126 vs. 109). In all three centres, the refusal rate was high at 54.5–64.9%.

**Conclusion:**

The HEW score represents a tool for the detailed recording and standardised reporting of potential organ donors and can enable homogenised reporting behaviour as a data basis for future improvement approaches.

## Einleitung

Im Jahr 2023 wurden in Deutschland bei 965 Menschen Organe nach dem Tod entnommen und 2877 Organe transplantiert, während es 10 Jahre zuvor 876 Organspender und 3035 gespendete Organe waren. Die Zahlen der realisierten Organspenden zeigen sich somit, trotz intensiver gesellschaftlicher Debatten zu diesem Thema, seit Jahren gleichbleibend bzw. in der Gesamtzahl der transplantierten Organe rückläufig [9]. Demgegenüber stehen 8700 Menschen, die auf eine lebensrettende Organtransplantation warten.

Die Ursachen des Rückganges an Organspendern sind bislang unklar, da der Großteil der Bevölkerung einer Organspende gegenüber positiv eingestellt ist und in den letzten Jahren sogar eine Zunahme der Spendebereitschaft postuliert wurde [8]. Als Grund für den Rückgang wird, neben einer mangelnden Zustimmung zur Organspende durch Dritte (Familienangehörige, Ehe‑/Lebenspartner), vor allem eine unzureichende Identifikation potenzieller Organspender angegeben [7]. Eine rezente Arbeit geht, auf Krankenhausleistungsdaten und Meldequoten an die Deutsche Stiftung Organspende (DSO) in den Jahren 2010–2015 basierend, von einer Zunahme der Anzahl potenzieller Spender von 13,9 % aus, gleichzeitig kam es aber zu einer Abnahme der Realisationsquote (realisierte Spenden/mögliche Spender) von 2,2 % [7]. Aus Sicht der Autoren liegt dies nicht an gesetzlichen Rahmenbedingungen, was sich auch im Scheitern der Einführungsversuche einer Widerspruchslösung ausdrückt. Als ursächlich für die abnehmende Realisationsquote und somit sinkenden Organspenderzahlen sehen Schulte et al. eine rückläufige und unzureichende Identifikation und Meldung potenzieller Spender an, die durch unterschiedliche Meldequoten bei eher vergleichbaren Realisierungsquoten in verschiedenen Krankenhäusern belegt werde. Intensive Debatten auf dieser Datenbasis führten zur Stärkung der Transplantationsbeauftragten und Einführung eines Organspenderegisters [2, 5]. Diese Anpassungen führten bislang jedoch noch nicht zur erhofften relevanten Steigerung der Organspenderzahlen, die zwischen 2021 und 2023 nahezu konstant blieb (933 vs. 965), während die Anzahl der gespendeten Organe sogar eine leicht rückläufige Tendenz zeigte (2905 vs. 2877; [9]). Auch die bislang noch ausstehende Initiierung des Organspenderegisters wird Analysen von Rithalia et al. zufolge allenfalls einen geringen Einfluss auf das Spendeaufkommen haben [3].

Wie von Schulte und Kollegen dargestellt unterscheidet sich die Kontaktquote, definiert als die Anzahl an Meldungen potenzieller Organspender an die DSO in Bezug auf die Gesamtzahl potenzieller Spender, erheblich und lag bei den untersuchten 6 Universitätsklinika zwischen < 5 % und ca. 25 % [7]. Diese Varianz ist zumindest anteilig ein Resultat der fehlenden Vorgabe, bei welchen Patienten der Kontakt zur DSO zwecks Meldung potenzieller Spender zu erfolgen hat, was die Verlässlichkeit der durchgeführten Hochrechnungen erheblich reduziert. So gibt es innerhalb der deutschen Universitätsklinika durchaus Unterschiede, welche Bedingungen im Hinblick auf irreversiblen Hirnfunktionsausfall (IHA) und Organspendewille erfüllt sein müssen, um eine Meldung an die DSO auszulösen.

Eine detaillierte Meldung von Verdachtsfällen anhand einheitlicher Meldekriterien würde hier die Möglichkeit bieten, eine verbesserte Datenbasis zur statistischen Untersuchung potenzieller Organspender zu erheben, um Handlungsmöglichkeiten und Ansatzpunkte zu identifizieren. In dieser Arbeit präsentieren wir mit dem Hirnfunktionsausfall-Eignung-Wille(HEW)-Score ein Werkzeug zur strukturierten Bewertung von potenziellen Organspendern und schlagen Kriterien zur einheitlichen Meldung potenzieller Spender an die DSO vor.

## Methoden

### Konsensbasierte Definition des HEW-Scores

Die Konsensuskonferenz der Transplantationsbeauftragten der Universitätsklinika in Nordrhein-Westfalen definierte die folgenden Variablen als Grundlage für eine Meldeentscheidung: IHA, Organspendewille und medizinische Eignung. Die Einzelausprägungen wurden für die Variable IHA in „IHA feststellbar“, „IHA erwartet/vermutet“ und „IHA nichtfeststellbar“ unterteilt. Für den Organspendewillen ergeben sich die Ausprägungen „Zustimmung“, „ungeklärt“ und „Ablehnung“. Die medizinische Eignung wird anhand des Vorliegens von Kontraindikationen (KI) zur Organspende mit den Ausprägungen „keine Kontraindikation“, „unklar“ und „KI bekannt“ erfasst. Die Konsensuskonferenz kam zu dem Schluss, dass die Meldung von potenziellen Organspendern an die DSO bei festgestelltem IHA in jedem Fall, bei erwartetem oder vermutetem IHA jedoch nur in Abwesenheit einer bekannten KI erfolgen sollte.

**Abb. 1:**
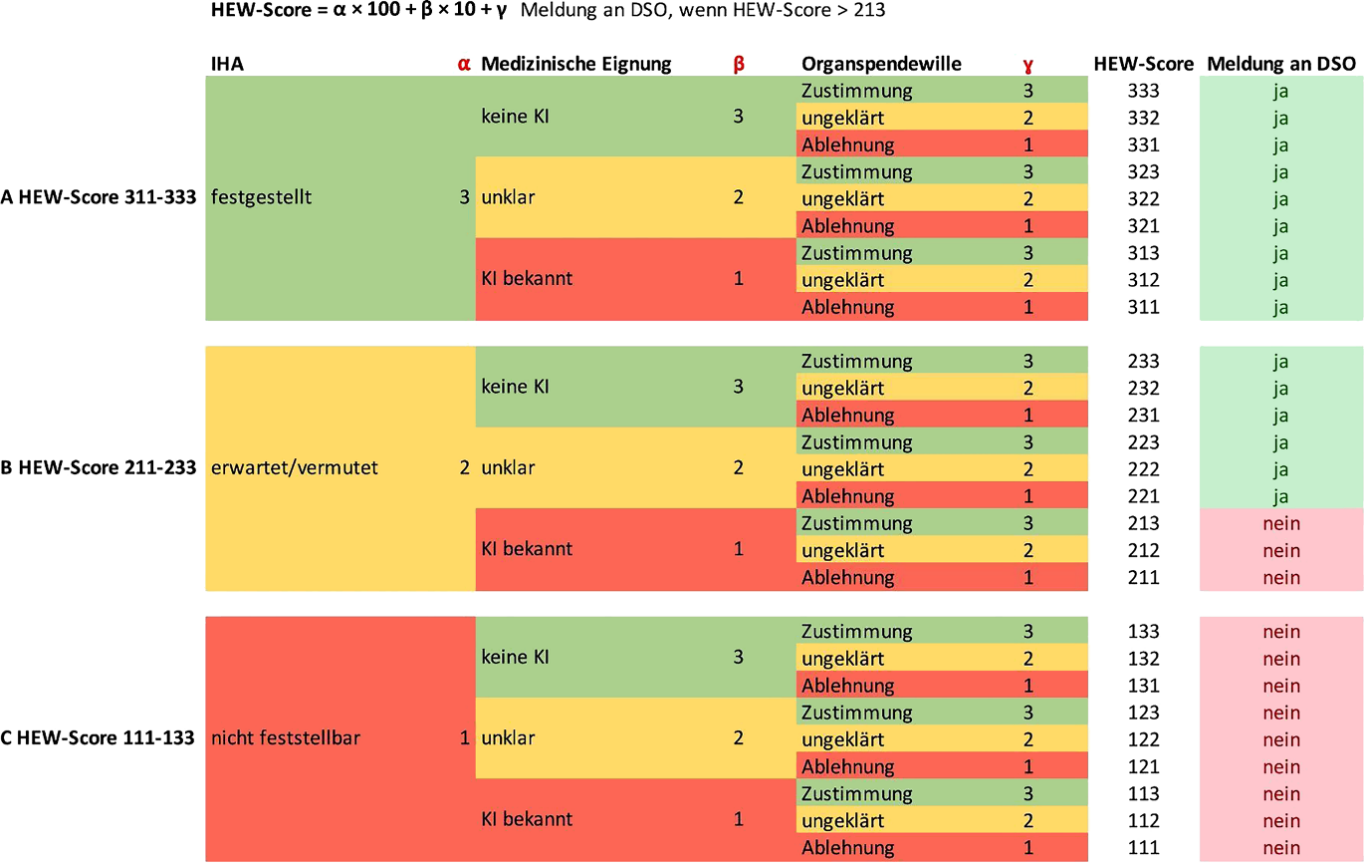
Berechnungsmatrix des HEW-Scores. Auf Basis der Einzelitems und des resultierenden Ergebnisses kann aus dem HEW-Score auch rückwirkend stets die einzelne Merkmalsausprägung nachvollzogen werden

Auf der Basis dieser Vorgaben vergaben wir für die einzelnen Faktoren die Werte 1–3 (1: nichterfüllt, 2: unklar, 3: erfüllt; „IHA“: ɑ[1; 2; 3], „medizinische Eignung“: β[1; 2; 3], „Organspendewille“: γ[1; 2; 3]). Der HEW-Score lässt sich mittels der Formel (ɑ × 100 + β × 10 + γ) berechnen (Abb. 1) und kann einen Wert zwischen 111 und 333 Punkten annehmen. Der Konsensuskonferenz zufolge sollte somit bei einem HEW-Score > 213 (d. h. ab 221) eine Meldung an die DSO erfolgen. Aufgrund sich ändernder Kenntnisse bez. Kontraindikationen o. ä. kann der HEW-Score auch mehrfach pro Patient erhoben werden. Es gilt dann der jeweils neueste Wert für die Meldung, die ggf. auch erst im Verlauf bei Überschreiten der Meldeschwelle erforderlich ist. Es ist daher nicht erforderlich die Eignung und den Willen des Patienten in direktem zeitlichem Zusammenhang mit dem IHA zu erfassen. Durch die Vergabe der Einzelwerte lässt sich auch retrospektiv rückschließen, welche Befundausprägung bei einem Patienten vorlag, ohne nochmals die Vorbefunde zu analysieren; z. B. entspricht ein HEW-Score von 223 der Konstellation „IHA erwartet, Kontraindikation ungeklärt, Spendewille bejaht“ (siehe Abb. 1).

### Datenerhebung und Stichprobe

Es erfolgte der retrospektive Einschluss aller Patienten, die über das Tool TransplantCheck im Jahr 2022 in den Universitätsklinika Bonn, Essen und Düsseldorf erfasst wurden (*n* = 907). Dieses Vorgehen wurde gewählt, um eine möglichst einheitliche und objektive Patientenauswahl zu gewährleisten. Für die eingeschlossenen Patienten wurde retrospektiv der individuelle HEW-Score ermittelt. Die vorliegende Studie wurde von der lokalen Ethikkommission der Universität Bonn genehmigt (Nr. 40/22).

**Abb. 2:**
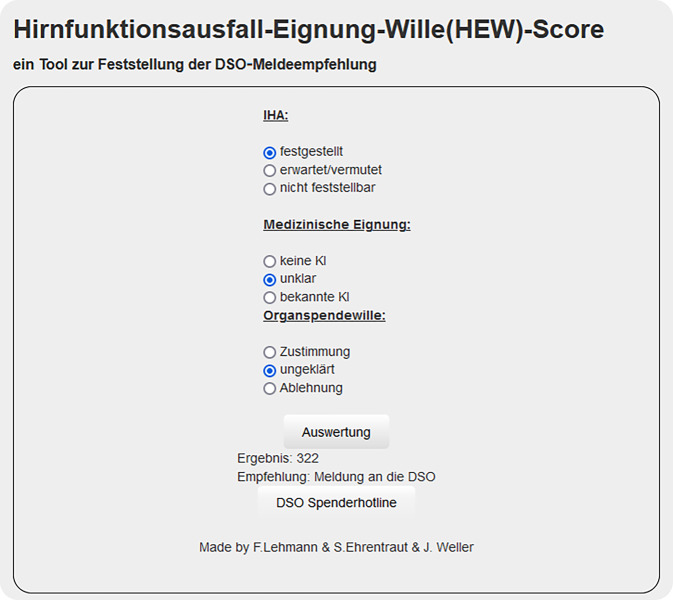
Beispielhafte Darstellung des Webinterface mit Auswertung und Ausgabe einer Meldeempfehlung basierend auf der Eingabe

Zur Ermittlung des HEW-Scores wurde entweder die Berechnungsmatrix verwendet (siehe Abb. 1) oder eine HTML-Eingabemaske, die Script-basiert den Score ermittelt (siehe e‑Supplement 1 und Abb. 2). Alternativ wäre eine Ermittlung mittels eines Tabellenkalkulationstools wie beispielsweise Excel (Microsoft, Redmond, WA, USA) über eine „WENNS-Funktion“ möglich:

=(WENNS(E2=„festgestellt“;3;E2=„erwartet“;2;E2=„vermutet“;1)*100)+(WENNS(E4=„keine KI“;3;E4=„unklar“;2;E4=„bekannte KI“;1)*10)+WENNS(E3=„Zustimmung“;3;E3=„ungeklärt“;2;E3=„Ablehnung“;1)

In diesem Beispiel müssten in den Zellen E2 bis E4 fest voreingestellte Eingabeoptionen, beispielsweise mittels der Funktion „Datenüberprüfung“ hinterlegt, werden.

Es erfolgte eine deskriptive Auswertung der anonymisierten aggregierten Daten mittels R (R Core Team, Version 4.2.1).

## Ergebnisse

**Abb. 3:**
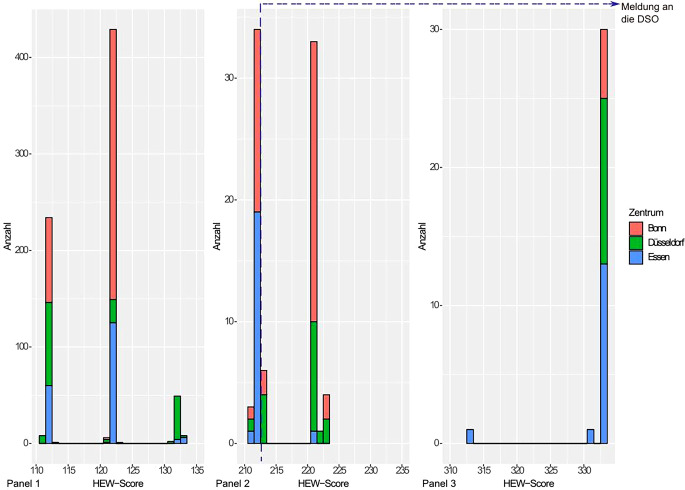
Häufigkeitsverteilung der HEW-Scores der Patienten des Jahrs 2022 aus den einzelnen Universitätsklinika. Die *gestrichelte Linie* stellt den Schwellenwert (213) dar, oberhalb dessen eine Meldung an die DSO empfohlen wird. Panel 1: Patienten, bei denen kein IHA eingetreten ist; Panel 2: Patienten mit vermutetem/erwartetem IHA; Panel 3: Patienten mit nachgewiesenem IHA

Im Jahre 2022 wurden von TransplantCheck 907 Fälle in den Unversitätsklinika Bonn, Düsseldorf und Essen erfasst und mittels HEW-Score bewertet. Hierbei ergab sich erwartungsgemäß eine überwiegende Anzahl an Fällen ohne nachgewiesenen IHA (*n* = 738), in denen es weder zu einer Klärung von eventuell vorhandenen Kontraindikation noch zur Ermittlung des Spenderwillens kam (Abb. 3).

Die Zahl der tatsächlich der DSO gemeldeten Fälle lag 13,5 % unter den gemäß HEW-Score zu meldenden Fällen (126 vs. 109; Tab. 1). Die Differenz ergab sich an den unterschiedlichen Standorten aus der retrospektiven Datenanalyse. Es handelte sich um Patienten mit eingetretenem IHA und bestehender Kontraindikation zur Spende (HEW 311–313) oder Ablehnung einer Spende (HEW xx 1).

**Tab. 1:** Anteil der tatsächlich im Jahr 2022 an die DSO gemeldeten Fälle und Anteil der Fälle mit einem HEW-Score > 213

Zentrum	Gemeldete Fälle; *n*	HEW > 213; *n* (%)	Abweichung; *n* (%)
Bonn	28	31 (7,4)	3 (9,7)
Düsseldorf	36	44 (20)	8 (18,2)
Essen	45	51 (19,1)	6 (11,8)
Gesamt	109	126 (13,9)	17 (13,5)

Abhängig vom verwendeten Cut-off-Wert ergibt sich bei Meldung aller Patienten mit möglichem oder erwartetem IHA (HEW > 133) eine Meldezahl von 169 Fällen, bei Verwendung des konsentierten Cut-off-Werts (HEW > 213) eine Meldezahl von 126 Fällen und bei Meldung aller Patienten mit nachgewiesenem IHA (HEW > 233) eine Meldezahl von 32 Fällen, entsprechend einer Schwankung um den Faktor 10 bzw. zwischen 1,2–11,6 % der Gesamtfälle.

Standortabhängig ließen sich Unterschiede im Vorgehen der 3 Zentren erkennen. Insbesondere bei erwartetem/vermutetem IHA wurden in Düsseldorf und Essen die Kontraindikationen auch bei fehlender Zustimmung des Patienten häufiger erhoben als in Bonn (17 [35 %] und 19 [40,9 %] vs. 5 [13,5 %]). In allen Standorten zeigte sich, dass für die Gruppe der möglichen Organspender (HEW > 211) die Ablehnungsrate hoch war (Düsseldorf 24 [64,9 %], Essen 37 [66,1 %], Bonn 24 [54,5 %]).

**Abb. 4:**
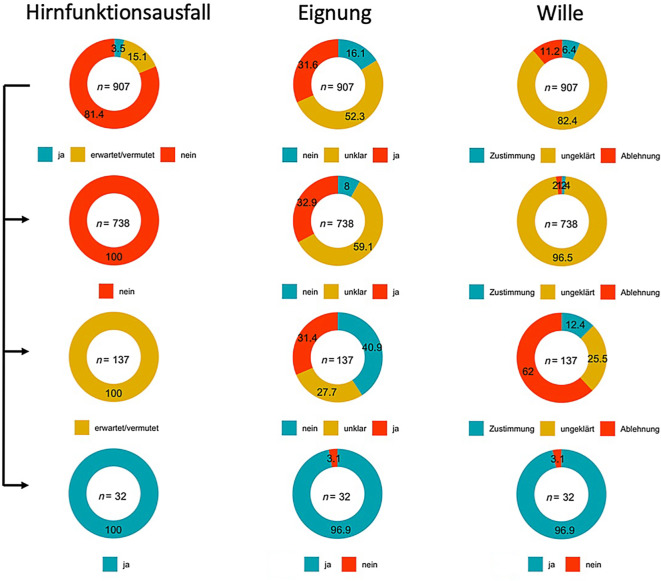
Aufteilung der einzelnen Fälle anhand des Merkmals IHA und der zusätzlichen Faktoren Eignung und Wille

Es zeigte sich, dass die aufwendige IHA-Diagnostik an allen Standorten überwiegend nur durchgeführt wurde, wenn weder Kontraindikationen noch eine Ablehnung vorlagen, was auch daran zu erkennen ist, dass in der Gruppe der Patienten mit diagnostiziertem IHA (HEW 3xx) an allen Standorten homogen Eignung und Zustimmung erfasst wurden. In Essen wurde nur in einem Fall hiervon abgewichen (Abb. 4). In den Fällen ohne nachgewiesenen IHA (*n* = 738, HEW 1xx) finden sich die Patienten, bei denen in der retrospektiven Analyse kein Anhalt für selbigen bestand. Bei einem Drittel dieser Patienten waren in dieser Analyse Kontraindikationen zu einer Organspende extrahierbar (z. B. bekanntes Tumorleiden), während der Wille zur Spende nur noch in Einzelfällen rekonstruierbar war. Es handelt sich damit um eine Kohorte, die aufgrund einer akuten, jedoch nichtrelevanten Hirnschädigung keinen IHA entwickelte und daher keine Kontaktaufnahme mit der DSO erfolgte.

## Diskussion

Die vorliegende Arbeit schlägt mit dem HEW-Score eine vereinheitlichte Form der Meldung potenzieller Organspender an die DSO vor. Die Analyse dieser standardisierten Daten kann womöglich weitere Erkenntnisse zu den auf niedrigem Niveau stagnierenden Zahlen der Organspenden in Deutschland liefern und potenzielle Ansatzpunkte identifizieren.

Auch 2023 setzt sich der Trend hin zu weniger Organspenden in Deutschland fort. Mit nur noch 11 Spendern pro 1 Mio. Einwohner stellt Deutschland weiterhin das Schlusslicht aller europäischen Länder dar und empfängt innerhalb des Eurotransplant-Raums mehr Organe, als es selbst abgibt.

Die Änderungen des Transplantationsgesetzes 2019 und 2020 [5] fokussierten sich hauptsächlich auf die Stärkung der Rolle des transplantationsbeauftragten Arztes in den Krankenhäusern. Eine Aufklärung der Patienten über die Organspende soll durch Hausärzte, hier mit einem Vergütungsanreiz (EBM-Ziffer 01480), und im Rahmen der vorgeschriebenen Erste-Hilfe-Kurse im Rahmen des Führerscheinerwerbs erfolgen. Zusätzlich sollen die Ausweisstellen von Bund und Ländern Aufklärungsmaterial und Organspendeausweise aushändigen. Ein weiterer Kernpunkt der damaligen Beschlüsse, die Einführung eines Registers zur Erfassung der persönlichen Entscheidung in Hinblick auf eine Organspende, ist zum gegenwärtigen Zeitpunkt noch im Aufbau und damit noch nicht funktional.

Im Rahmen der damaligen Debatte führte die Annahme, dass es eine große Zahl an nichtdetektierten, potenziellen Organspendern gebe, zu den genannten Änderungen. Basis dieser Betrachtung waren Daten von Schulte et al. [7], in denen, ausgehend von einer Schätzung, ca. 10 % der potenziellen Organspender auch hätten spenden müssen. Dies hätte eine Quote von ca. 30 Spenden pro 1 Mio. Einwohner zur Folge gehabt. Die Analyse unserer Daten unter Berücksichtigung verschiedener Cut-off-Werte für die DSO-Meldung potenzieller Spender (HEW > 133, HEW > 213, HEW > 233) zeigt eine Schwankung der Meldezahlen um den Faktor 10 und verdeutlicht, dass Hochrechnungen basierend auf Kontakt- (DSO-Kontakte/potenzielle Spender) oder Konversionsquoten (Organspenden/DSO-Kontakte) ohne die Definition einer präzisen Meldeschwelle nur eingeschränkt aussagekräftig sind. Die standardisierte Erfassung potenzieller Organspender und vor allem die Schwelle zur Meldung an die DSO ermöglicht daher aus Sicht der Autoren in Zukunft aussagekräftigere Analysen.

Die Gründe für die weiterhin sinkende Anzahl an Organspenden in Deutschland sind aus Sicht der Autoren somit, anders als von Schulte et al. postuliert, nicht ausschließlich in einer unzureichenden Identifikation potenzieller Organspender zu suchen. Auch wenn es während der COVID-Pandemie einen, letztlich auch erwartbaren, Rückgang gab, blieb dennoch eine Trendwende nach Anpassung der Richtlinie zur Feststellung des irreversiblen Hirnfunktionsausfalls durch die Bundesärztekammer aus.

In der vorliegenden Arbeit bestätigt sich erneut, dass die Ablehnungsquote im Rahmen des Organspendeprozesses hoch ist [4]. Dies ist insbesondere der Fall, wenn die Entscheidung durch Angehörige getroffen werden muss [4], was aus Sicht der Autoren die Notwendigkeit unterstreicht, eine individuelle Entscheidung zu Lebzeiten zu unterstützen.

Bereits 2012 zeigte das von der DSO beauftragte In-house-Koordinationsprojekt [1], dass die unterdurchschnittliche Spenderquote in Deutschland nicht durch eine unzureichende Meldung potenzieller Spender allein erklärbar ist. Dennoch ergibt es natürlich prinzipiell Sinn, Werkzeuge zu etablieren, die eine rechtzeitige Identifikation von potenziellen Organspendern ermöglichen, und das In-house-Koordinationsprojekt zeigte, dass die im Projekt etablierten Maßnahmen zu einer Stabilisierung der Organspendezahlen der beteiligten Krankenhäuser führten. Das DETECT-Tool [6] stellt in diesem Zusammenhang beispielsweise eine potenzielle und leicht zu implementierende Lösungen zur automatisierten Meldung potenzieller Organspender dar. Die retrospektive Auslegung des Projekts erlaubte jedoch keine weiteren Rückschlüsse. Insbesondere in Krankenhäusern mit einem hohen Spenderpotenzial oder einer unterdurchschnittlichen Melderate könnte *gerade eine Kombination des HEW-Scores mit dem DETECT-Tool dazu beitragen*, niederschwellig potenzielle Spender zu identifizieren.

Die vorliegende Zusammenfassung ist hinsichtlich der erfassten Zahlen und des Patientenkollektivs dem Selektionsbias der eingeschlossenen Maximalversorger unterliegend. Aufgrund der Tatsache, dass die beteiligten Zentren an der Erstellung der Meldematrix beteiligt waren, ist eine hohe Adhärenz naheliegend. Dennoch erscheint es hinsichtlich der Transparenzsteigerung und Vereinfachung der DSO-Meldung – insbesondere für Krankenhäuser mit geringer Erfahrung in der Spenderidentifikation – relevant, einfache, transparente und einheitliche Meldekriterien zu definieren. Erst nach Einführung eines bundesweit einheitlichen Verfahren zur Meldung potenzieller Organspender erscheint die Vergleichbarkeit der Ablehnungs- und Konversionsquoten zwischen verschiedenen Krankenhäusern gegeben.

## Schlussfolgerung

Mithilfe des HEW-Scores und der dargestellten Werkzeuge kann die Meldeschwelle potenzieller Spender an die DSO standardisiert und transparent definiert werden. Hierdurch kann eine verbesserte Datengrundlage zur Beurteilung des tatsächlichen Meldeverhaltens in Deutschland geschaffen werden.

## Supplementary Information

E‑Supplement 1 Java-Code zur Erstellung eines Webfrontends zur Nutzung des HEW-Scores und Link auf das GitHub Repository mit der aktuellsten Code Versione.

## Data Availability

Die erhobenen Datensätze können auf begründete Anfrage in anonymisierter Form beim korrespondierenden Autor angefordert werden.
