# Supplementary material for: HEW score—a tool for the homogenisation of donor registrations to the DSO: Multicentre retrospective analysis of three university hospitals
Source: Med Klin Intensivmed Notfmed. 2025 Jan 28;120(8):653–60. [Article in German] doi: 10.1007/s00063-024-01237-6 (PMC12594715; doi:10.1007/s00063-024-01237-6)
Supplement: Supplementary file 1 — E‑Supplement 1 Java-Code zur Erstellung eines Webfrontends zur Nutzung des HEW-Scores und Link auf das GitHub Repository mit der aktuellsten Code Versione. [file 63_2024_1237_MOESM1_ESM.pdf]

E-Supplement 1

Der in der Publikation verwendete Code für die Erstellung des Webfrontends zur Nutzung und Implementierung des HEW Scores, steht unter einer MIT Lizenz in folgendem GitHub Repository zur Verfügung.

https://stefanehrentraut.github.io/HEW-Score/

In der Version von 12-2024 lautete der verwendete Java-Code wie folgt:

```
<!DOCTYPE html>
<html lang="de">
  <head>
       <meta charset="UTF-8">
      <meta name="viewport" content="width=device-width, initial-
scale=1.0">
        <title>Hirnfunktionsausfall-Eignung-Wille (HEW)-
Score</title>
    <style>
        /* Globale Schriftarten und Basis-Layout */
      body {
        font-family: Arial, sans-serif;
        margin: 0;
        padding: 0;
        background-color: #f4f4f4;
      }

      /* Container für den Rechner */
      #calculator {
        max-width: 600px; /* Maximale Breite */
        margin: 20px auto;
        padding: 20px;
        background-color: white;
        border: 1px solid #ddd;
        border-radius: 10px;
        box-shadow: 0 0 10px rgba(0, 0, 0, 0.1);
        box-sizing: border-box;
      }

      /* Flexbox-Layout für die Eingabefelder */
      .section {
        display: flex;
        flex-direction: column;
        margin-bottom: 20px;
      }

      .section p {
        margin: 10px 0;
```

```
          font-size: 1.1em;
        }

        /* Anpassungen für die Eingabefelder */
        input[type="radio"] {
          margin-right: 10px;
        }

        /* Stil für den Auswertungsbutton */
        button {
          background-color: #4e7ea8;
          color: white;
          padding: 12px 20px;
          border: none;
          border-radius: 5px;
          cursor: pointer;
          font-size: 1em;
          transition: background-color 0.3s;
          width: 100%;
          box-sizing: border-box;
        }

        button:hover {
          background-color: #345d7e;
        }

        /* Responsives Design: für kleinere Geräte (z.B. Smartphones)
*/
        @media (max-width: 600px) {
          #calculator {
            padding: 15px;
          }

          button {
            font-size: 1.1em;
          }

          .section p {
            font-size: 1em;
          }
        }

        /* Stil für das Ergebnis */
        #result {
          margin-top: 20px;
          font-weight: bold;
          font-size: 1.2em;
        }
```

```
      /* Stil für den Footer */
      footer {
        text-align: center;
        font-size: 0.9em;
        margin-top: 30px;
        color: #666;
      }

      footer a {
        color: #4e7ea8;
        text-decoration: none;
      }

      footer a:hover {
        text-decoration: underline;
      }
    </style>
  </head>
  <body>
    <h1>Hirnfunktionsausfall-Eignung-Wille (HEW)-Score</h1><h3> ein
Tool zur Feststellung der DSO Meldeempfehlung</h3>
    <div id="calculator">
      <div>
        <p><strong>IHA:</strong></p>
        <input type="radio" id="IHA6" name="IHA" value="300">
festgestellt<br>
        <input type="radio" id="IHA3" name="IHA" value="200">
erwartet/vermutet<br>
        <input type="radio" id="IHA0" name="IHA" value="100"> nicht
feststellbar<br>
             <br>
        <p><strong>Medizinische Eignung:</strong></p>
        <input type="radio" id="medizinischeEignung3"
name="medizinischeEignung" value="30"> keine KI<br>
        <input type="radio" id="medizinischeEignung2"
name="medizinischeEignung" value="20"> unklar<br>
        <input type="radio" id="medizinischeEignung1"
name="medizinischeEignung" value="10"> bekannte KI<br>
        <p><strong>Organspendewille:</strong></p>
        <input type="radio" id="organspendewille06"
name="organspendewille" value="3"> Zustimmung<br>
        <input type="radio" id="organspendewille03"
name="organspendewille" value="2"> ungeklärt<br>
        <input type="radio" id="organspendewille01"
name="organspendewille" value="1"> Ablehnung<br>

        <br>
```

```
      </div>
       <br>
      <button onclick="calculate()">Auswertung</button>
       <br><br> <!-- Leerzeile einfügen -->
      <div id="result"></div>
      <p>
    <button
onclick="location.href='https://dso.de/organspende/fachinformationen
/organspendeprozess/24h-spendermeldung'">DSO
Spenderhotline</button></p>
<br>
<p>Made by S.F. Ehrentraut, F. Lehmann & J. Weller <br><br> siehe
"Der HEW-Score - Ein Werkzeug zur Homogenisierung der
Spendermeldungen an die DSO. Eine multizentrisch retrospektive
Analyse dreier Universitätsklinika." <a
href="https://link.springer.com/article/ 10.1007/s00063-024-01237-
6"> Medizinische Klinik - Intensivmedizin und Notfallmedizin
2024</a></p>
    <p>Weitere Informationen zum medizinischen Hintergrund finden
Sie in der <a href="https://github.com/stefanehrentraut/HEW-
Score#readme" target="blank">Readme</a></p></div>
      <p>
    Copyright (c)  S.F. Ehrentraut, F. Lehmann, J. Weller 2024.
<br><br>Dieser Code steht unter der <a
href="https://github.com/StefanEhrentraut/HEW-
Score/blob/main/LICENSE" target="blank">MIT-Lizenz</a>.
    Bitte nennen Sie die ursprünglichen Autoren bei Nutzung,
Abwandlung oder Weiterentwicklung des Codes.
</p>

    <script>
      function calculate() {
        var iha = parseFloat(
          document.querySelector('input[name="IHA"]:checked').value
        );
        var organspendewille = parseFloat(

document.querySelector('input[name="organspendewille"]:checked').val
ue
        );
        var medizinischeEignung = parseFloat(

document.querySelector('input[name="medizinischeEignung"]:checked').
value
        );
        var result = (iha + organspendewille + medizinischeEignung);
        var resultMessage =
```

```
          result > 213 ? "Empfehlung: Meldung an die DSO" :
"Empfehlung: Keine Meldung an die DSO";
        document.querySelector("#result").innerHTML =
          "Ergebnis: " + result + "<br>" + resultMessage;
      }
    </script>
  </body>
</html>
<!--Dieses Projekt basiert auf dem Originalcode von [F. Lehmann,
S.F. Ehrentraut und J. Weller/Universitätsklinikum Bonn],
erfügbar unter [https://stefanehrentraut.github.io/HEW-Score/] Die
Autoren sind entsprechend der Lizenzbedingungen bei Nutzung,
Abwandlung und Weiterentwicklung anzugeben.!-->
```
